# Comparative transcriptomics of aphid species that diverged > 22 MYA reveals genes that are important for the maintenance of their symbiosis

**DOI:** 10.1038/s41598-023-32291-3

**Published:** 2023-04-01

**Authors:** Jacob A. Argandona, Dohyup Kim, Allison K. Hansen

**Affiliations:** 1 Department of Entomology, University of California, Riverside, 900 University Ave, Riverside, CA 92521 USA; 2 Stanford University School of Medicine, 291 Campus Drive, Stanford, CA 94305 USA

**Keywords:** Evolution, Genetics

## Abstract

Most plant-sap feeding insects have obligate relationships with maternally transmitted bacteria. Aphids require their nutritional endosymbiont, *Buchnera aphidicola*, for the production of essential amino acids. Such endosymbionts are harbored inside of specialized insect cells called bacteriocytes. Here, we use comparative transcriptomics of bacteriocytes between two recently diverged aphid species, *Myzus persicae* and *Acyrthosiphon pisum,* to identify key genes that are important for the maintenance of their nutritional mutualism. The majority of genes with conserved expression profiles in *M. persicae* and *A. pisum* are for orthologs previously identified in *A. pisum* to be important for the symbiosis. However, asparaginase which produces aspartate from asparagine was significantly up-regulated only in *A. pisum* bacteriocytes, potentially because *Buchnera* of *M. persicae* encodes its own asparaginase enzyme unlike *Buchnera* of *A. pisum* resulting in *Buchnera* of *A. pisum* to be dependent on its aphid host for aspartate. One-to-one orthologs that explained the most amount of variation for bacteriocyte specific mRNA expression for both species includes a collaborative gene for methionine biosynthesis, multiple transporters, a horizontally transmitted gene, and secreted proteins. Finally, we highlight species-specific gene clusters which may contribute to host adaptations and/or accommodations in gene regulation to changes in the symbiont or the symbiosis.

## Introduction

The establishment and maintenance of a mutualistic relationship between a eukaryotic host and a microbe involves a series of regulatory changes for both the host and microbe^1^. These regulatory changes facilitate the integration of both the host’s and the microbe’s physiologies for the successful exchange of symbiotic goods. One of the best studied models of insect-endosymbiont mutualistic interactions is the pea aphid, *Acyrthosiphon pisum*, and its obligate endosymbiont bacterium, *Buchnera aphidicola*. This integrated host-symbiont metabolism has been well characterized biochemically and genetically, identifying which enzymes are present and/or expressed by the aphid and its symbiont, *Buchnera*^2–6^. Within specialized aphid cells that harbor *Buchnera*, called bacteriocytes, the aphid’s metabolism is integrated with *Buchnera* for the production of essential amino acids^5,6^. Aphid host genes that have been identified to play a role in the regulation and maintenance of this mutualistic relationship involve transporters, a cycle (the GS/GOGAT pathway) that incorporates waste ammonia into amino acids through glutamate, and genes that complement *Buchnera*’s essential amino acid pathways^5–7^. Nevertheless, what regulatory mechanisms are conserved and lineage specific between different aphid species is still unclear.

Similar to *A. pisum*, the green peach aphid, *Myzus persicae*, belongs to the *Macrosiphini* tribe within the family Aphididae: Aphidinae^8^ and is estimated to have diverged from *A. pisum* ~ 22 million years ago^9^. While both aphid species are pests and have similar lifecycles their host plant range varies dramatically^10^. For example, *A. pisum* is acknowledged as a specialist of *Fabaceae* host plants, where sympatric aphid populations are divided into a number of different biotypes that specialize on specific *Fabaceae* species^11^. In contrast, *M. persicae* differs from *A. pisum* in that it is a true generalist herbivore and can feed on 40 different plant families including many economically and agriculturally important crop species^12^. Furthermore, rapid transcriptional plasticity of multigene families has allowed *M. persicae* clones to colonize up to 100 species of host plants without genetic specialization^10^. Given this transcriptional plasticity in response to diverse host plant diets, it is of interest to compare the regulation of bacteriocytes between a closely related aphid specialist and generalist, *A. pisum* and *M. persicae,* respectively.

To further understand how aphid gene regulation has evolved to support its symbiotic relationship with *Buchnera* in bacteriocytes we conducted an interspecies comparative transcriptomics approach between *M. persicae* and *A. pisum.* Here we identify conserved and lineage specific mechanisms of aphid host regulation between *M. persicae* and *A. pisum* in bacteriocytes when both aphid species develop in a common environment and feed on the same host plant species, *Vicia fava,* in which both species clones here display high fitness on^13–15^.

## Results

### Differential gene expression analysis of symbiosis genes for each aphid species

Total high-quality paired-reads that mapped to the *M. persicae* genome for bacteriocyte and body samples were an average of ~ 24,022,544 and 37,852,850 reads, respectively (Supplemental Table 1). Total high-quality paired-reads that mapped to the *A. pisum* genome for bacteriocyte and body samples were an average of ~ 34,163,080 and 34,083,851 reads, respectively (Supplemental Table 1). A total of 5097 genes were significantly differentially expressed (i.e. FDR adjusted p-values were ≤ 0.05 with 1.5 fold change (FC)) in *M. persicae* bacteriocytes compared to body tissues (Supplemental Tables 2 and 3), where a total of 1986 and 3111 genes were significantly up-regulated and down-regulated, respectively (Supplemental Table 3). For *A. pisum*, a total of 7325 genes were significantly differentially expressed in *A. pisum* bacteriocytes compared to body samples (Supplemental Tables 4 and 5), where a total of 3205 and 4120 genes were significantly up-regulated and down-regulated, respectively (Supplemental Table 5).

**Figure 1:**
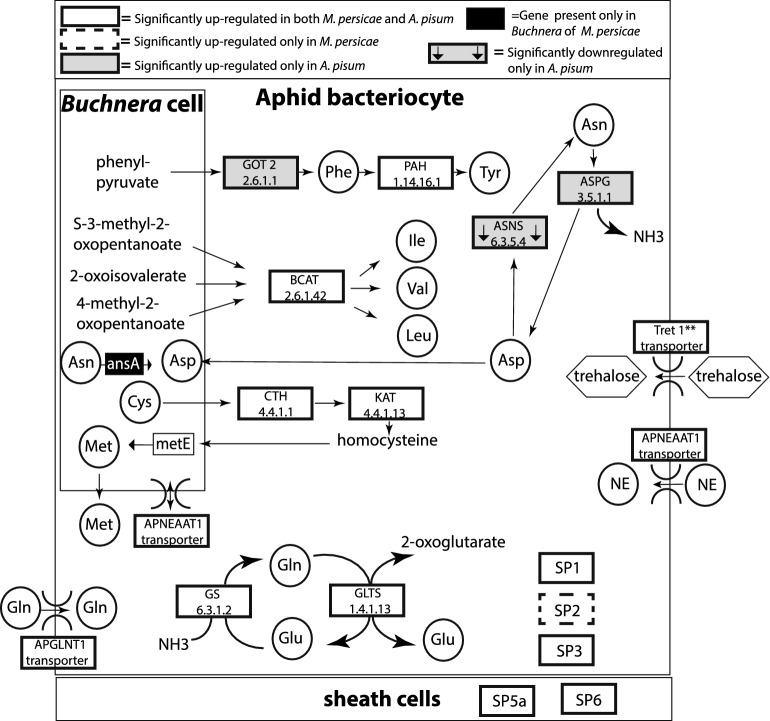
Gene expression of aphid genes involved in the integrative metabolism with *Buchnera*. ** indicates four homologs significantly up-regulated in both *M. persicae* and *A. pisum*.

The expression of aphid genes in bacteriocytes that were previously identified in *A. pisum* to be collaborative in the biosynthesis of *Buchnera*’s essential amino acids^5,6^ were examined here for both aphid species (Table 1, Fig. 1). When comparing both *M. persicae* and *A. pisum,* six orthologs displayed the same expression pattern where five orthologs were significantly up-regulated in bacteriocytes compared to body tissues (Branched chain amino acid transaminase (*BCAT*), Phenylalanine 4-monooxygenase (*PAH*), l-cysteine-S-conjugate thiol-lyase, Cystathionine gamma-lyase (*CTH*), and Homocysteine S-methyltransferase) and one ortholog was significantly down-regulated in bacteriocytes compared to body tissues for both species (Aspartate transaminase, *GOT1*) (Table 1, Fig. 1). Species-specific expression patterns for collaborative genes in *M. persicae* bacteriocytes compared to body samples include the up-regulation of Threonine ammonia-lyase, whereas this ortholog was not significantly up-regulated in *A. pisum* (Table 1, Fig. 1). For *A. pisum* species-specific expression patterns for bacteriocytes compared to body samples include the up-regulation of Aspartate transaminase (*GOT2*), whereas this ortholog was not significantly up-regulated in *M. persicae* (~ 1.44 FC) (Table 1, Fig. 1).

**Table 1:** Expression of collaborative essential amino acid aphid orthologs in bacteriocytes compared to body tissues for two different aphid species. Each row contains a shared ortholog between *M. persicae* and *A. pisum* except for N/A which indicates no ortholog was identified between species

E.C. number	Gene name^1^	Pathway	*Myzus persicae*			*Acyrthosiphon pisum*		
			Gene ID^2^	FDR	LogFC	Gene ID^2^	FDR	LogFC
4.3.1.19	Threonine ammonia-lyase	Ile	**g3539**	0.004	1.86	100165866	0.881	− 0.07
2.6.1.42	**Branched chain amino acid transaminase, *BCAT***	Ile, Val, Leu	**g15579**	0.000	2.20	**100167587**	0.000	2.96
2.6.1.1	Aspartate transaminase, *GOT2*	Phe, Tyr	g12035	0.072	0.53	**100144899**	0.000	3.21
2.6.1.1	**Aspartate transaminase, *GOT1***	Phe, Tyr	**g15167**	0.001	− 2.05	**100163139**	0.000	− 3.56
2.6.1.1	Aspartate transaminase, *GOT1*	Phe, Tyr	N/A	N/A	N/A	100161812	nd	nd
2.6.1.1	Aspartate transaminase, *GOT1*	Phe, Tyr	g15165	nd	nd	N/A	N/A	N/A
2.6.1.1	Aspartate transaminase, *GOT1*	Phe, Tyr	g14986	0.282	0.39	100165255	0.399	0.31
1.14.16.1	**Phenylalanine 4-monooxygenase, *PAH***	Tyr	**g26128**	0.000	4.85	**100166971**	0.000	4.85
4.4.1.1	**Cystathionine gamma-lyase; *CTH***	Met	**g10610**	0.000	5.57	**100159197**	0.000	6.48
4.4.1.1	Cystathionine gamma-lyase	Met	g10608	0.185	0.68	**100159560**	0.001	− 1.16
4.4.1.13	**L-cysteine-S-conjugate thiol-lyase**	Met	**g6762**	0.000	2.69	**100164839**	0.000	3.96
2.1.1.10	**Homocysteine S-methyltransferase**	Met	**g3958**	0.000	3.04	**100168557**	0.000	3.26
2.1.1.10	Homocysteine S-medfthyltransferase	Met	g9831	0.719	0.22	100159972	0.680	− 0.11

^1^Bolded Gene names indicate orthologs in both aphid species are significantly differentially expressed between bacteriocytes compared to body tissues where both FDR p-value $\le$ 0.05 and fold change (FC) $\ge$ 1.5 (i.e. LogFC $\ge$ 0.5849)).

^2^Bolded Gene IDs indicate that the aphid gene is significantly differentially expressed between bacteriocytes compared to body tissues in that aphid species. nd= not detected.

Other important aphid genes that were previously identified to be important for *A. pisum*’s symbiosis with *Buchnera* include genes that are involved in the recycling of ammonia, biosynthesis of amino donors or intermediates for *Buchnera*’s essential amino acid pathways, transporters for amino acids or trehalose, and novel secreted proteins^5–7,14,16–18^ (Table 2). When comparing both *M. persicae* and *A. pisum* 13 orthologs were significantly up-regulated in bacteriocytes compared to body tissues for both species and include enzymes involved in the GS/GOGAT cycle (Glutamate synthase (*GLTS*) and Glutamate-ammonia ligase (*GS2*)), Ornithine aminotransferase (*OAT*), the Amino acid transporters (*ApGLNT1*) and (*ApNEAAT1*), four Trehalose transporters (*TRET1*), and four secreted proteins such as SP1, SP3, SP5a, and SP6. Only two orthologs were significantly down-regulated in bacteriocytes compared to body tissues for both species, secreted protein SP4 and Aspartate transaminase (*GOT1*) (Tables 1 and 2; Fig. 1). Species-specific expression patterns in *M. persicae* bacteriocytes compared to body tissues include the up-regulation of only one gene, secreted protein SP2, whereas this ortholog was not significantly differentially expressed in *A. pisum* (Table 2, Fig. 1). Multiple genes in *A. pisum* displayed species-specific up-regulation in bacteriocytes compared to body tissues for an additional homolog of Glutamate-ammonia ligase (*GS2*) in *A. pisum*, Asparaginase, the Mitochondrial 2-oxoglutarate/malate carrier, and an additional homolog of the Trehalose transporter in *A. pisum* (Table 2, Fig. 1).

**Table 2:** Expression of symbiosis-related aphid orthologs in bacteriocytes compared to body tissues in two different aphid species. Each row contains a shared ortholog between *M. persicae* and *A. pisum* except for N/A which indicates no ortholog was identified between species.

		Pathway	*Myzus persicae*			*Acyrthosiphon pisum*		
E.C. number	Gene name^1^		Gene ID^2^	FDR	LogFC	Gene ID^2^	FDR	LogFC
Amino acid enzymes								
1.4.1.14	**Glutamate synthase (*GLTS*)**	Glu	**g22515**	0.000	2.04	**100158883**	0.000	2.07
6.3.1.2	**Glutamate-ammonia ligase (*GS2*)**	Glu	**g15463**	0.000	1.89	**100160139**	0.000	1.48
6.3.1.2	Glutamate-ammonia ligase	Glu	N/A	N/A	N/A	**100165282**	0.007	4.60
6.3.1.2	Glutamate-ammonia ligase	Glu	g8271	0.401	0.81	N/A	N/A	N/A
2.6.1.13	Ornithine aminotransferase (*OAT*)	Glu	**g6537**	0.029	-1.35	N/A	N/A	N/A
2.6.1.13	**Ornithine aminotransferase**	Glu	**g6576**	0.000	3.21	**100168809**	0.000	4.00
3.5.1.1	Asparaginase	Asn	g14033	0.131	0.48	**100158730**	0.000	-1.52
3.5.1.1	Asparaginase	Asn	g8195	0.142	0.71	**100164179**	0.000	5.32
6.3.5.4	Asparagine synthetase (*ASNS*)	Asn	g23256	0.142	1.05	**100160265**	0.022	-0.86
Transporters								
	**Amino acid transporter (*ApGLNT1*)**		**g19243**	0.000	2.38	**100159667**	0.000	1.40
	**Amino acid transporter (*ApNEAAT1*)**		**g25548**	0.000	1.38	**100168251**	0.017	0.82
	Mitochondrial 2-oxoglutarate/malate carrier		g11494	0.063	0.81	**100159664**	0.000	3.34
	Trehalose transporter (*TRET1*)		g27282	0.093	0.58	**100159441**	0.000	1.28
	**Trehalose transporter (*TRET1*)**		**g22841**	0.000	3.64	**100165626**	0.000	5.28
	**Trehalose transporter (*TRET1*)**		**g11139**	0.000	5.19	**100169458**	0.000	7.54
	**Trehalose transporter (*TRET1*)**		**g16824**	0.000	4.90	**100161021**	0.000	7.09
	**Trehalose transporter (*TRET1*)**		**g12940**	0.000	3.70	**100169115**	0.000	3.14
Secreted proteins								
	***SP1***		**g23997**	0.000	5.78	**100167607**	0.000	7.94
	*SP2*		**g21195**	0.042	1.53	100158873	0.19	-0.54
	***SP3***		**g18732**	0.000	5.24	**100164129**	0.000	7.37
	*SP4*		**g14959**	0.009	-1.41	**100169357**	0.000	-3.07
	***SP5a***		**g19669**	0.031	1.06	**100163734**	0.000	2.47
	***SP6***		**g11563**	0.000	3.67	**100160550**	0.000	3.71

^1^Bolded Gene names indicate orthologs in both aphid species are significantly differentially expressed between bacteriocytes compared to body tissues where both FDR p-value $\le$ 0.05 and fold change (FC) $\ge 1.5$ (i.e. LogFC $\ge$ 0.5849).

^2^Bolded Gene IDs indicate that the aphid gene is significantly differentially expressed between bacteriocytes compared to body tissues in that aphid species. nd= not detected.

The differential expression of lineage specific gene clusters was also examined for both *M. persicae* and *A. pisum* (Supplemental Table 6)*.* Here we define “*A. pisum* lineage specific genes” as genes that are not present in *M. persicae* (non- *M. persicae* genes) and likewise “*M. persicae* lineage specific genes” are genes that are not present in *A. pisum* (non- *A. pisum* genes). Moreover, we define “conserved genes” here as homologs that are present in both *A. pisum* and *M. persicae.* Out of 1053 lineage specific gene clusters in *M. persicae* (consisting of 4622 genes) a total of 376 genes were significantly differentially expressed between bacteriocytes compared to body tissues. Approximately, 42 percent of these latter genes were significantly up-regulated in bacteriocytes compared to body tissues and include genes primarily associated with gene regulation such as DNA and RNA binding proteins, and uncharacterized genes (Supplemental Table 7). For *A. pisum*, 580 lineage specific gene clusters were identified (consisting of 1689 genes), and a total of 289 genes from these clusters were significantly differentially expressed between bacteriocytes compared to body tissues (Supplemental Table 8). Approximately, 22% of these latter genes were significantly up-regulated in bacteriocytes compared to body tissues and include genes primarily associated with Balbiani ring genes, death-associated inhibitor of apoptosis 1-like genes, UDP-glucuronosyltransferase genes, lysosomal and kelch associated genes, heat shock genes, ribosomal proteins, and hypothetical genes. (Supplemental Table 8).

### KEGG pathway analysis

Gene Set Enrichment Analysis (GSEA) identified seven and two shared pathways in both *M. persicae* and *A. pisum* that were significantly positively and negatively enriched, respectively, in bacteriocytes compared to body tissues (Table 3). The positively enriched pathways display hallmarks of metabolically active cells as these pathways are involved in DNA replication, RNA processing, and protein turnover (Table 3). In contrast, negatively enriched pathways that were shared between aphid species were primarily involved in cell signaling and adhesion (Table 3). Species-specific patterns in the enrichment of KEGG pathways were noted where seven and 13 KEGG pathways were significantly positively enriched in only *M. persicae* or *A. pisum* bacteriocytes compared to body tissues, respectively, and one and seven KEGG pathways were significantly negatively enriched in only *M. persicae* or *A. pisum* bacteriocytes compared to body tissues, respectively (Supplemental Table 9).

**Table 3:** GSEA analyses of both aphid species (Mp= *M. persicae* and Ap= *A. pisum*) displaying all KEGG pathways that were significantly enriched in bacteriocytes compared to body for both species.

KEGG pathway significantly enriched in bacteriocyte	Mp NES^1^	Ap NES^1^
**Positively enriched**		
Ribosome	1.941	2.842
Mismatch repair	1.922	1.861
Glycine, serine, and threonine metabolism	1.755	1.607
DNA replication	1.738	2.151
Cysteine and methionine metabolism	1.688	1.348
Proteasome	1.649	2.060
Nucleotide excision repair	1.556	1.759
**Negatively enriched**		
Neuroactive ligand-receptor interaction	− 1.880	− 1.593
Cell adhesion molecules	− 1.700	− 1.523

^1^*NES* normalized enrichment score; Significance = the normalized p ≤ 0.05 and FDR q ≤ 0.25.

### Inter-species comparison of one-to-one orthologs

**Figure 2:**
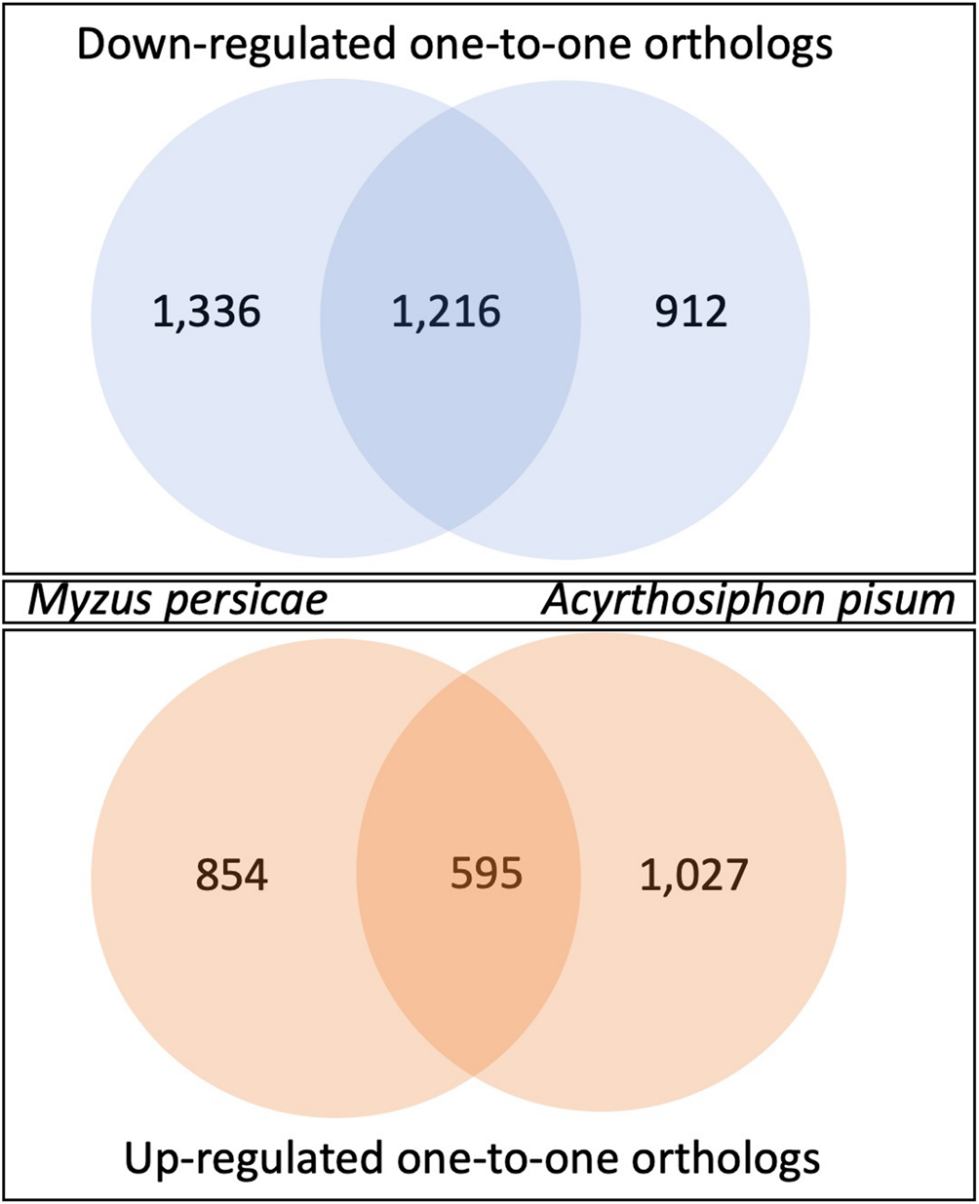
Overview of one-to-one orthologs that have significant differential gene expression in *M. persicae* and *A. pisum* for bacteriocytes compared to body tissues, where significance of up- or down-regulation between tissue types is determined for genes with an FDR corrected p-value $\le$ 0.05 and fold change (FC) $\ge$ 1.5 (i.e. LogFC $\ge$ 0.5849). The shared portion of Venn diagrams indicate that the one-to one ortholog was significantly down-regulated (top panel) or up-regulated (bottom panel) in both aphid species. Un-shared portions of Venn diagrams indicate that the ortholog was only down-regulated (top panel) or up-regulated (bottom panel) in one of the aphid species (left side *M. persicae*; right side *A. pisum*).

**Figure 3:**
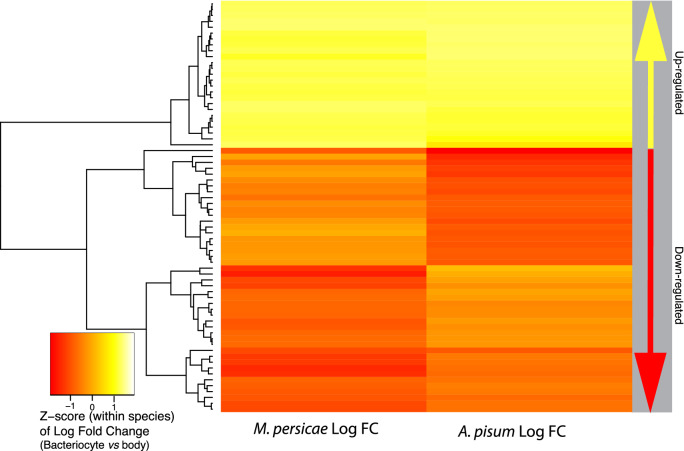
Inter-species ortholog comparison of the top 70 one-to-one orthologs with the greatest variance in standardized Log fold-change expression of bacteriocytes compared to body tissues for both *M. persicae* and *A. pisum*. “Up-regulated” on the right panel indicates up-regulated in bacteriocytes compared to body tissue and “Down-regulated” indicates down-regulated in bacteriocytes compared to body tissue. Each row represents a shared one-to-one ortholog between *M. persicae* and *A. pisum*. See detailed ortholog gene numbers and GO terms in Supplemental Fig. S1.

**Figure 4:**
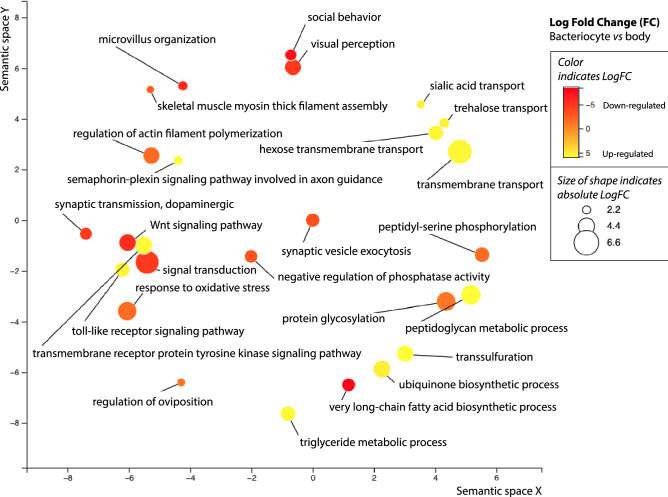
Approximately 54% of the one-to-one orthologs identified in inter-species ortholog analysis have GO term annotations, which are represented as circles in the figure (see Supplemental Table S10 for detail). Multidimensional scaling (MDS) was used in Revigo to reduce the dimensionality of the matrix for these 38 GO terms based on pairwise similarities in biological function. The size of the circle and color indicates the Log fold change of the ortholog (bacteriocyte compared to body) within a unique GO term category.

A total of 9465 shared one-to-one orthologs were identified between *M. persicae* and *A. pisum* and ~ 23% of these orthologs were significantly expressed in bacteriocytes compared to the body tissue for both aphid species (Fig. 2; Supplemental Table 6). To determine which of these differentially expressed orthologs have the biggest effect in fold-change between bacteriocytes and body for both species similar to Korb et al.^19^ and Georgiadou et al.^20^ we conducted PCA where axis 1 and 2 explained ~ 53.3% and 46.7% of the variance in the data, respectively. The top 100 shared orthologs (70 were non-redundant between both axis 1 and 2) with the highest positive and negative correlations with principal components 1 and 2 consist of 25 up-regulated and 45 down-regulated orthologs (Fig. 3, Supplemental Table 10). Symbiosis related genes that were up-regulated in both *M. persicae* and *A. pisum* for this subset of orthologs include Cystathionine gamma-lyase (*CTH*), the secreted proteins SP1 and SP3, the horizontally transferred gene *rlpA*, and two facilitated Trehalose transporter *TRET1* genes (Table 4). Other annotated genes up-regulated in both include multiple acetyl-coenzyme A transporters along with other transporter genes, receptors such as draper and plexins, and the E3 ubiquitin-protein ligase *MARCH2* (Table 4). Based on dendrogram clustering of standardized logFC in bacteriocytes compared to body tissues for these 70 paired-orthologs in *M. persicae* and *A. pisum*, up-regulated orthologs appear to have very similar logFC profiles (Fig. 3, Supplemental Fig. 1). In contrast, down-regulated orthologs appear to have two distinct logFC profiles with a subset of orthologs having either greater or lower logFC magnitudes in *M. persicae* or *A. pisum* (Fig. 3, Supplemental Fig. 1). For example, multiple orthologs that were associated with visual perception were down-regulated at a higher magnitude in *M. persicae* and orthologs associated with an amino acid transporter, heat shock protein, and peroxidase like protein were down-regulated at a higher magnitude in *A. pisum* (Supplemental Fig. 1, Table 10). The only GO term significantly enriched for up-regulated orthologs was GO:0055085 for transmembrane transport (p-value = 0.00027) (Fig. 4). Two GO terms were significantly enriched for down-regulated orthologs and include GO:0007601 for visual perception (p-value = 0.00043) and GO:0005524 for ATP binding (p-value = 0.00098) (Fig. 4).

**Table 4:** The 25 aphid ortholog proteins that were identified with PCA as having the greatest magnitude of up-regulation in the bacteriocyte compared to the body for both aphid species**.**

Mp gene	Ap gene ID	NCBI protein name	Mp LogFC	Ap LogFC
g23997	100167607	**SP1**	5.78	7.94
g16265	100163669	**RlpA family protein-like precursor**	5.74	7.53
g10610	100159197	**cystathionine gamma-lyase isoform 1**	5.57	6.48
g11338	100160627	**protein draper isoform X1**	5.55	7.06
g25444	100568670	**plexin A3**	5.49	7.63
g7735	100169049	uncharacterized protein LOC100169049 isoform X1	5.48	2.53
g22717	100166357	**organic cation transporter protein**	5.40	7.55
g7417	100575173	**plexin-A3**	5.40	7.60
g18732	100164129	**SP3**	5.25	7.37
g20745	100168678	**neprilysin-21 isoform X1**	5.23	7.02
g11139	100169458	**facilitated trehalose transporter Tret1**	5.19	7.33
g18635	100167419	**acetyl-coenzyme A transporter 1**	5.18	7.88
g13201	100164813	**major facilitator superfamily domain-containing protein 6 isoform X1**	5.14	6.19
g15906	100164131	**acetyl-coenzyme A transporter 1 isoform X1**	5.12	4.95
g11347	100161188	**leucine-rich repeat-containing protein 15**	5.08	6.15
g13798	100160909	uncharacterized protein LOC100160909 precursor	5.01	7.05
g24975	100162203	uncharacterized protein LOC100162203	4.99	6.63
g26344	100168152	**sialin**	4.96	5.86
g19207	100169490	**acetyl-coenzyme A transporter 1**	4.92	7.31
g16824	100161021	**facilitated trehalose transporter Tret1**	4.90	7.89
g2999	100569384	**E3 ubiquitin-protein ligase MARCH2-like isoform X1**	4.90	6.36
g16263	100570509	uncharacterized protein LOC100570509	4.87	7.67
g27533	100164029	**decaprenyl-diphosphate synthase subunit 2**	4.83	6.80
g16264	100570300	uncharacterized protein LOC100570300	4.72	8.00
g16266	100165005	uncharacterized protein LOC100165005 precursor	4.38	8.02

All proteins in the table were significantly up-regulated in each aphid species (*Mp* *M. persicae* and *Ap* *A. pisum*) where FDR adjusted p-values were ≤ 0.05 with ≥ 1.5-fold change (FC). Bolded proteins indicate those that are characterized with annotations in NCBI.

## Discussion

The aphid bacteriome, the organ housing the intracellular symbiont *Buchnera* and the location of the integrated essential amino acid metabolism, is functionally, structurally, and developmentally conserved among nearly all aphids^21–24^. Here, we interrogated gene expression profiles of these symbiotic host cells in two aphid species that diverged ~ 22 MYA and detected extensive expression conservation in addition to key species-specific expression changes between *A. pisum* and *M. persicae*. The species-specific gene expression changes we detected between both species may contribute to host adaptations and/or accommodations in gene regulation to changes in the symbiont or the symbiosis.

A consequential expression difference we detected between *A. pisum* and *M. persicae* bacteriocytes is the significant up-regulation (~ 40X) of Asparaginase (3.5.1.1) only in *A. pisum* bacteriocytes (Table 2; Fig. 1). This enzyme is critical for the hosts’ integrated metabolism with *Buchnera* as Asparaginase converts asparagine, which is one of the most abundant non-essential amino acids in the aphid’s sap-diet^13^, into aspartate and ammonia. *Buchnera* the nutritional endosymbiont of *A. pisum* is located inside of bacteriocytes and requires aspartate from the aphid host, because it cannot make it *de novo*. Further *Buchnera* needs aspartate for the biosynthesis of the essential amino acids lysine and threonine, which the aphid and *Buchnera* require for survival^5^. We predict that this expression difference is because the *Buchnera* endosymbionts of *M. persicae* unlike *Buchnera* of *A. pisum* encode their own asparaginase gene (*ansA*); *ansA* is the only additional essential amino acid biosynthetic gene found in *Buchnera M. persicae* that is absent in *Buchnera A. pisum*^25^ (Fig. 1). In consequence, *Buchnera* of *M. persicae* is not entirely dependent on its aphid host for the biosynthesis of aspartate, whereas *Buchnera* of *A. pisum* is dependent on its aphid host for access to this important amino acid. It will be of interest of future studies to determine if *A. pisum* can regulate *Buchnera*’s production of lysine and threonine by fine-tuning aspartate availability through the aphid encoded Asparaginase and/or the aphid Asparagine synthetase (*ASNS*) enzyme that converts aspartate back into asparagine. For example, *ASNS* is significantly down-regulated only in *A. pisum* indicating that *A. pisum* bacteriocytes are in high demand for aspartate biosynthesis during this lifestage and environmental condition (Fig. 1).

The overwhelming majority of genes with conserved expression profiles in *M. persicae* and *A. pisum* are for orthologs previously identified in *A. pisum* to be important for the nutritional symbiosis (Tables 1 and 2). For example, in both *M. persicae* and *A. pisum*, similar bacteriocyte expression profiles were found for five aphid orthologs *BCAT*, *PAH*, *CTH*, l-cysteine-S-conjugate thiol-lyase, and Homocysteine S-methyltransferase) which were up-regulated in bacteriocytes and complement five of *Buchnera*’s essential amino acid pathways (isoleucine, valine, leucine, tyrosine, and methionine) (Table 1, Fig. 1). Other notable genes that were previously recognized as important in the *A. pisum*- *Buchnera* symbiosis that were up-regulated in both *A. pisum* and *M. persicae* bacteriocytes here include ornithine aminotransferase (*OAT*) providing a potential intermediate for *Buchnera*’s arginine biosynthesis^5^, the amino acid transporters *ApGLNT1* and *ApNEAAT1* that are important in amino acid transport for the symbiosis^7,17^, Trehalose transporters (*TRET1*), which may be crucial in providing *Buchnera* with glucose after trehalose is transported into the bacteriocytes and subsequently converted into glucose^14,18,26^, and the GS/GOGAT cycle, which is hypothesized to up-grade ammonia into amino donors to help fuel the nitrogen limited nutritional symbiosis^5^. However, one aphid ortholog (*GOT1*), which collaborates with *Buchnera* in the biosynthesis of the amino acid phenylalanine, was only significantly up-regulated in *A. pisum* bacteriocytes. *GOT* up-regulation in bacteriocytes convergently occurs in other independently evolved Hemipteran symbioses including the mealybug^27^, psyllid^28^, and whitefly^29^. Therefore, it is possible that in another environmental/host plant diet condition the up-regulation of *GOT1* in *M. persicae* would be less variable in expression and would be significantly up-regulated in bacteriocytes. Alternatively, an uncharacterized enzyme in *M. persicae* or *Buchnera* of *M. persicae* may be responsible for this enzymatic step.

We further identified that bacteriocytes of both *A. pisum* and *M. persicae* are metabolically highly active, because mRNA, protein, and DNA replication KEGG pathways were significantly positively enriched in aphid bacteriocytes (Table 3). Our positive enrichment of DNA replication genes in bacteriocytes support recent results from Nozaki and Shigenobu who found a dramatic increase in the number of chromosome sets per bacteriocyte cell (i.e., ploidy) as *A. pisum* ages after live birth into the reproductive viviparous adult stage^30^. Ploidy of insect chromosomes is a general feature of bacteriocytes in *A. pisum*^30^, *M. persicae*^31^, and other diverse insect taxa (reviewed in^30^). Here we also found that the Glycine, Serine, and Threonine Metabolism and the Cysteine and Methionine Metabolism were positively enriched in bacteriocytes which potentially indicates a high demand for not only the essential amino acids methionine and cysteine but also for methyl group donors for DNA methylation during the DNA replication process. In support of this hypothesis, Pers and Hansen^18^ found that DNA methylation occurs in bacteriocytes throughout aphid development (1st instar-reproductive adult stage) where *A. pisum* bacteriocytes are more heterogenous in methylation profiles for the 1st and 2nd instars compared to subsequent lifestages suggesting that there is a high demand for methyl donors via both de novo methylation and maintenance methylation early and in subsequent lifestages, respectively, throughout nymphal and young adult development.

Unique gene expression profile differences between *M. persicae* and *A. pisum* bacteriocytes were primarily highlighted by the expression of species-specific gene clusters between *M. persicae* and *A. pisum*. These profile differences indicate that there is a species-specific difference in the chromosome metabolism and the apoptotic pathway. For example, NCBI annotated Balbiani ring paralogs, are lineage specific in *A. pisum,* and consist of 54 paralogous gene copies that are composed of ankyrin repeat domains*.* Six of these paralogs are significantly enriched in *A. pisum* bacteriocytes compared to body tissues (Supplemental Table 8). Interestingly, Kwak et al.^32^ determined that 52 of the copies represented recent, rapid expansions of this gene cluster in *A. pisum* and homologs of this gene cluster were not found in *M. persicae* or any of the other Hemipteran species examined in that study, except for *Aphis glycines* (Supplemental Tables 6 and 8). Based on functional studies in *Drosophila*, Balbiani ring genes are involved in polytene chromosomes, which are commonly found in fly salivary glands where homologous chromosome copies in the nucleus are aligned and attached to each other, and chromatin of active genes unfold in loops forming puffs^33^. At this time, it is unclear if polytene chromosomes occur in *A. pisum* or other aphids. Moreover, it is unclear if these Balbiani ring genes have similar functions in polytene chromosome formation, or alternatively if these *A. pisum* genes have evolved new functions. This mode of DNA replication, however, can result in advantages for specialized cell types like bacteriocytes, which have a high demand for gene expression. For example, in polytene chromosomes gene amplification can occur for targeted genes because re-replication only occurs for sub-sets of the DNA and under-replication can occur for loci that are targeted for down-regulation^34^. The trade-off of polytene chromosomes however is an increase in DNA double-strand breaks and errors that can result in the up-regulation of the apoptotic machinery^34^. Interestingly, a significant positive enrichment for the homologous recombination pathway, a DNA double-strand break repair pathway was only identified in *A. pisum* bacteriocytes and not *M. persicae* (Supplemental Table 9).

Regarding apoptotic machinery, the apoptotic pathway was recently annotated and characterized in *A. pisum*^35^. Here we found that one of the four effector caspases (Ap-ICE-2) were significantly up-regulated in bacteriocytes compared to body tissues in *A. pisum*, however none of the other caspases including the initiator caspases or the adaptor protein were significantly differentially expressed at this lifestage and tissue comparison for *A. pisum* (Supplemental Table 5). Lopes et al.^35^ predict that the duplicated copies of caspases in *A. pisum* may have redundant functions, or alternatively may have evolved new functions that are related or unrelated to apoptosis. None of the latter caspase orthologs in *M. persicae* were differentially expressed, however the adapter protein (g10569) was significantly down-regulated in *M. persicae* bacteriocytes (Supplemental Tables 3 and 6). Five *A. pisum* bacteriocyte-specific inhibitors of apoptosis (IAPs) were also characterized in Lopes et al.^35^ to have bacteriocyte-specific expression in the adult stage of *A. pisum* and displayed an antiapoptotic role *in vivo* in a *Drosophila* model. Here in *A. pisum* we found one of the five latter genes significantly up-regulated in 4th instar bacteriocytes compared to body tissue (Ap-Deterin-1) (Supplemental Table 5). The one-to-one ortholog of Ap-Deterin-1 in *M. persicae* (g14656) was also up-regulated significantly here (Supplemental Tables 3 and 6). The up-regulation of this latter IAP in both *A. pisum* and *M. persicae* may suggest a conserved role in inhibiting apoptosis in nymphal (4th instar) bacteriocytes and not just the adult stage of aphids. When examining lineage specific apoptosis-related *A. pisum* genes, which are not encoded in *M. persicae,* we found that six previously characterized IAPs that belong to paralogy group C (Ap-IAP-C9; Ap-IAP-C14; Ap-IAP-C15; Ap-IAP-C17; Ap-IAP-C18; Ap-IAP-C21) Lopes et al.^35^ and one “putative inhibitor of apoptosis” gene (115034293), based on BLAST, are significantly up-regulated in bacteriocytes compared to body tissues in *A. pisum* (Supplemental Table 8). At this time the role of lineage specific IAP genes from group C in *A. pisum* is unknown.

Novel secreted protein transcripts that are unique to aphids, such as *SP1*, *SP3*, *SP5a*, and *SP6*^16^, were significantly up-regulated in both aphid species (Table 2). The functional role of these orphan genes with N-terminal signal sequences is not fully understood however they may enter the secretory pathway, and SP1 and SP3 are expressed in bacteriocytes and SP5a and SP6 are expressed in the sheath cells surrounding the bacteriocytes^16^. Interestingly, our comparative transcriptomics analysis with PCA identified both *SP1* and *SP3* within the top 70 one-to-one orthologs that explain the greatest amount of variation in fold change for all one-to-orthologs that were significantly differentially expressed in bacteriocytes (N = 2188) for both *M. persicae* and *A. pisum* (Table 4). The gene SP1 in *M. persicae* was found to be regulated in bacteriocytes via miR-92a using a dual luciferase assay^36^, indicating that miRNA::mRNA interactions may be very important for the regulation of aphid genes that are involved in the aphid-*Buchnera* mutualism. Other symbiosis genes that fell into this subset of the top 70 one-to-one orthologs included the collaborative gene, *CTH*, which is involved in methionine biosynthesis with *Buchnera* (Fig. 1), two trehalose transporter orthologs, and the horizontally transferred gene *rlpA* (^37^; Table 4). Nakabachi et al.^37^ demonstrated that RplA is synthesized as a protein in the maternal bacteriocyte and is transported into *Buchnera* cells. This horizontally transmitted gene is hypothesized to produce lytic translgycosylase for *Buchnera* cell wall remodeling and is largely omnipresent in Aphidoidea unlike other horizontally transferred genes that were lost in certain aphid lineages^38^.

In summary, when controlling for environmental factors, including the same host plant species, both conserved and lineage specific patterns of gene expression were identified in bacteriocytes from *M. persicae* (clone Fava) and *A. pisum* (clone LSR1). Since both aphid species display high fitness on this host plant species (*V. fava*)^13,15^, and display similar developmental times, at least for the aphid clones used in previous studies and here^15,39^ we do not predict that bacteriocyte gene expression profiles are due to a stress response. Collaborative genes involved in the nutritional symbiosis were largely conserved between *M. persicae* and *A. pisum* however the differential expression of Asparaginase between *M. persicae* and *A. pisum* may signify differences in aspartate production in both the aphid bacteriocyte and in *Buchnera*. Moreover, pathway and lineage specific gene cluster differences between *A. pisum* and *M. persica*e in this study may signify species-specific adaptations, including the putative use of polytene chromosomes in *A. pisum* for the strategic amplification and down-regulation of genes for symbiosis, whereas species-specific genes up-regulated in *M. persica*e bacteriocytes were primarily composed of proteins containing DNA and RNA regulatory domains. It will be of interest for future studies to determine how plastic bacteriocyte gene expression responses are between aphid species using additional aphid clones in ecology related studies when they feed on different host plant diets, and are exposed to different environmental conditions.

## Materials and methods

### Insect rearing and RNA sequencing

To control for environmental variation both species, *M. persicae* and *A. pisum,* were allowed to develop to 4th instar and fed on the same host plant species, *Vicia faba*, at 20 °C with a 16:8-h light-dark cycle with ~ 30–40% humidity in Intellus Ultra controller Percival incubators (Percival Scientific, Inc., Perry, IA, USA). Transcriptomic data produced previously for *A. pisum* using these latter conditions in Kim et al.^14^ was used for this study. Both studies however were performed around the same time period and collected by the same individual for DK’s Ph.D thesis. For *M. persicae,* a genetically homogenous strain of *M. persicae* (Sulzer) from Medina-Ortega and Walker^40^ was divided into three sub-lines for this study and has been reared on *V. faba* stably with high vigor for > 5 years in culture (clone Fava). Briefly, similar to *A. pisum* (clone LSR1) in Kim et al.^14^ *M. persicae* was divided into three sub-lines and was reared on *V. fava* (23 ± 2 days after germination (~ 5 whorls)) for over 20 generations at 20 °C with a 16:8-h light-dark cycle in Intellus Ultra controller Percival incubators (Percival Scientific, Inc., Perry, IA, USA). Approximately 200 aphids that were at 4th instar were dissected from each sub-line to co-collect both bacteriocytes with *Buchnera* (N = 3 sublines/biological replicates per species) and other body cells without *Buchnera* (N = 3 sublines/biological replicates per species) using the same methods detailed in Kim et al.^14^. Developmental times for *A. pisum* and *M. persicae* on *V. fava* at the detailed conditions stated above were similar to Pers and Hansen^39^ for *A. pisum* and Hong et al.^15^ for *M. persicae*, where both aphid species were at 4th instar on day six. Briefly, pooled total RNA of bacteriocytes and body cells were extracted using the *Quick*-RNA Microprep kit (Zymo Research, Irvine, CA, USA). Extracted RNA samples were treated with DNase I and purified with the RNA Clean & Concentrator kit (Zymo Research). Strand-specific RNA-seq libraries were generated with poly-A enrichment and sequenced on 1 lane of an Illumina HiSeq 4000 (Illumina, San Diego, CA) with paired-end 150bp reads (see Supplemental Methods for more detail). Reads for all RNA-Seq samples were submitted to the Sequence Read Archive of the National Center for Biotechnology Information (NCBI) under BioProject ID PRJNA866154.

The following RNAseq pipeline was conducted for both *A. pisum*^14^ and *M. persicae* raw RNAseq reads and followed the code detailed in “Dataset_S9_RNAseq_Code” from Pers and Hansen^18^ (see Supplemental Methods for detail on entire pipeline). First, sequenced RNA reads were quality-checked with FASTQC v.0.11.9^41^ and reads were trimmed using Trimmomatic v.0.39^42^. The trimmed reads were aligned using HISAT2 v.2.2.1^43^ against the chromosomal assemblies of clone AL4f for *A. pisum*^44^ and *M. persicae* clone O v2^9^. The mapped reads for each gene were quantified as raw read counts using StringTie v.2.2.1^45^, visualized using Principal Components Analyses (PCA) (Supplemental Fig. 2) in R/4.2.0^46^, and differential expression of transcripts between bacteriocytes and body cells was determined in R/4.2.0^46^ using edgeR v.3.28.0 with the exact test^47^. Similar to Kim et al.^14^ statistical significance for differentially expressed genes was determined if FDR adjusted p-values were ≤ 0.05 with 1.5-fold change (FC), indicated as “logFC” = log2 fold change between the groups. For KEGG pathway analysis of aphid genes^9,44^, KO numbers were retrieved using KofamKoala^48^ and Gene Set Enrichment Analysis (GSEA)^49^ was used to determine which KEGG pathways were positively or negatively enriched at the normalized p ≤ 0.05 and FDR q ≤ 0.25., as described in Pers and Hansen^18^.

### Identifying orthologous clusters

Orthologous clusters of proteins shared between *A. pisum*^44^ and *M. persicae*^9^ were identified using default settings in OrthoVenn2^50^ using the longest protein isoforms (see Supplemental Methods for detail). One-to-one orthologs were examined between *A. pisum* and *M. persicae* for PCA (see below), and the enrichment of GO terms for orthologs was determined using OrthoVenn2 see Supplemental Methods for detail^50^. All unshared protein clusters that were unique to *A. pisum* or *M. persicae* were examined further for differential gene expression analyses (see above). Proteins were annotated using NCBI annotations for *A. pisum* clone AL4f and by screening for matches to the NCBI nr database (downloaded on 06/2022) for *M. persicae* clone O v2 using DIAMOND v.2.0.13^51^ with an e value cutoff of 10e-10.

### Comparative transcriptomics using principal component analysis

To compare patterns of gene expression between species without species-specific variation, specifically focusing on genes associated with symbiosis that are differentially expressed in the bacteriocyte compared to body tissues, we conducted interspecies comparative transcriptomics analysis using PCA following the pipeline of Georgiadou et al.^20^ (see Supplemental Methods for detailed pipeline on interspecies transcriptomics analyses). Pcord (version 4.25)^52^ was used for PCA analysis. Similar to Korb et al.^19^ we obtained the top 50 genes that contributed the most to each principal component axis (axes 1 and 2) by identifying orthologs with the top negative (e.g. 25 genes) and positive (e.g. 25 genes) correlations from the principal components output loading matrix. We then examined the annotations for each ortholog protein (see above) from these top 100 orthologs. To display a heatmap of the logFC for one-to-one orthologs SeqCode’s^53^ HeatMapper was used and Revigo^54^ was used to visualize and summarize ortholog GO terms (see Supplemental Methods for more detail).

## Supplementary Information

Supplementary Information 1.

Supplementary Tables.

## Data Availability

Raw RNA sequencing data are available at NCBI under SRA BioProject ID: PRJNA866154 under SAMN30154147, SAMN30154148, SAMN30154149, SAMN30154150, SAMN30154151, SAMN30154152.
