# Supplementary material for: Comparative transcriptomics of aphid species that diverged > 22 MYA reveals genes that are important for the maintenance of their symbiosis
Source: Sci Rep. 2023 Apr 1;13:5341. doi: 10.1038/s41598-023-32291-3 (PMC10067822; doi:10.1038/s41598-023-32291-3)

Supplemental Methods

Library preparation: Strand-specific and barcode indexed RNA-seq libraries were generated from 300ng total RNA for each sample. Poly-A enrichment and library prep was done using the KAPA mRNA Stranded library preparation kit (KK8421, Kapa Biosystems, Cape Town, South Africa), following the instructions of the manufacturer. Libraries were amplified with 12 cycles of PCR. The fragment size distribution of the libraries was verified via micro-capillary gel electrophoresis on a Bioanalyzer 2100 (Agilent, Santa Clara, CA). The libraries were quantified by fluorometry on a Qubit fluorometer (LifeTechnologies, Carlsbad, CA) and pooled in equimolar ratios. The pool was quantified by qPCR with a Kapa Library Quant kit (Kapa Biosystems) and sequenced on 1 lane of an Illumina HiSeq 4000 (Illumina, San Diego, CA) with paired-end 150bp reads.

RNAseq bioinformatics pipeline: It is important to note that the transcriptome data in Kim et al. [1] was not analyzed on fava separately as bacteriocyte versus body but instead included the host-plant treatment for alfalfa in the model as well. In turn, a standardized pipeline that compared just fava samples of *A. pisum* was used here for both *A. pisum* [1] and *M. persicae*, where raw RNAseq reads followed the code detailed in "Dataset_S9_RNAseq_Code" from Pers and Hansen [2]. First, sequenced RNA reads were quality-checked with FASTQC v.0.11.9 [3], and adapters and low-quality reads were trimmed using Trimmomatic v.0.39 [4] with the following parameters: ILLUMINACLIP:combined.fna:2:30:10 LEADING:3 TRAILING:3 SLIDINGWINDOW:4:15 MINLEN:36. The trimmed reads were aligned using HISAT2 v.2.2.1 [5] against the chromosomal assemblies of clone AL4f for *A. pisum* [6] downloaded from NCBI on 7/2022 and *M. persicae* clone O v2 [7] downloaded on 6/2022 from Zenodo. The mapped reads for each gene were quantified as raw read counts using StringTie v.2.2.1 [8] using gff files annotated for each aphid species [6,7]. Differential expression of transcripts between bacteriocytes and body cells was determined in R/4.2.0 [9] using edgeR v.3.28.0 with the exact test [10].

Ortholog analysis: Orthologous clusters of proteins shared between *A. pisum* [6] and *M. persicae* [7] were identified using default settings in OrthoVenn2 [11] on 7/2022, which assigns orthologs based on OrthoMCL [12]. One-to-one orthologs were examined between *A. pisum* and *M. persicae* for Principal Components Analyses (PCA) (see below), and the enrichment of GO terms for orthologs was determined using OrthoVenn2, which tests for significance using the hypergeometric distribution [11]. All unshared protein clusters that were unique to *A. pisum* or *M. persicae* were examined further for differential gene expression analyses (see above). Proteins were annotated using NCBI annotations for *A. pisum* clone AL4f and by screening for matches to the NCBI nr database (downloaded on 06/2022) for *M. persicae* clone O v2 using DIAMOND v.2.0.13 [13] with an e value cutoff of 10e-10.

Interspecies transcriptomics analysis pipeline: Similar to Georgiadou et al. [14] to compare patterns of gene expression between species without species-specific variation, specifically focusing on genes associated with symbiosis that are differentially expressed in the bacteriocyte compared to body tissues, we conducted comparative transcriptomics using PCA. Following Georgiadou et al. [14] we

identified 1:1 orthologs between both aphid species (see above) and selected all orthologs that displayed significant differential gene expression within each aphid species for bacteriocytes compared to body tissue samples with an average logFC >0.5849 or logFC < -0.5849. To allow comparison of the relative magnitude of changes in gene expression between *A. pisum* and *M. persicae*, orthologs for each species were ranked in descending order of absolute logFC and each gene was given a value of 100 divided by rank and then multiplied by the sign of the original logFC. These values were then input into PCA to highlight which orthologs in both *A. pisum* and *M. persicae* shared the most similar and dissimilar gene expression profiles in the bacteriocytes compared to body tissues. Pcord (version 4.25) [15] was used for PCA analysis using the correlation coefficients cross-products matrix and default settings as suggested in McCune & Mefford [16]. Similar to Korb et al. [17] we obtained the top 50 genes that contributed the most to each principal component axis (axes 1 and 2) by identifying orthologs with the top negative (e.g. 25 genes) and positive (e.g. 25 genes) correlations from the principal components output loading matrix. We then examined the annotations for each ortholog protein (see above) from these top 100 orthologs. To display a heatmap of the logFC for one-to-one orthologs SeqCode's [18] HeatMapper was used with dendrogram clustering of ortholog LogFC with Z-score conversion within species. SeqCode Heatmapper calls the R heatmap.2 function (gplots package) with the parameter distfun = dist, which by default adopts the Euclidean distance (method = "euclidean") for clustering [18]. Revigo [19] was used to visualize and summarize ortholog GO terms.

References:

1. Kim, D., Minhas, B. F., Li-Byarlay, H. & Hansen, A. K. Key Transport and Ammonia Recycling Genes Involved in Aphid Symbiosis Respond to Host-Plant Specialization. *G3 GenesGenomesGenetics* **8**, 2433–2443 (2018).
2. Pers, D. & Hansen, A. K. The boom and bust of the aphid's essential amino acid metabolism across nymphal development. *G3 GenesGenomesGenetics* **11**, (2021).
3. Andrews, S. FastQC: A Quality Control Tool for High Throughput Sequence Data.

   www.bioinformatics.babraham.ac.uk/projects/fastqc/ (2010).

4. Bolger, A. M., Lohse, M. & Usadel, B. Trimmomatic: a flexible trimmer for Illumina sequence data. *Bioinformatics* **30**, 2114–2120 (2014).
5. Kim, D., Langmead, B. & Salzberg, S. L. HISAT: a fast spliced aligner with low memory requirements. *Nat. Methods* **12**, 357–360 (2015).
6. Li, Y., Park, H., Smith, T. E. & Moran, N. A. Gene Family Evolution in the Pea Aphid Based on Chromosome-Level Genome Assembly. *Mol. Biol. Evol.* **36**, 2143–2156 (2019).
7. Mathers, T. C. *et al.* Chromosome-Scale Genome Assemblies of Aphids Reveal Extensively Rearranged Autosomes and Long-Term Conservation of the X Chromosome. *Mol. Biol. Evol.* **38**, 856–875 (2020).

8. Pertea, M. *et al.* StringTie enables improved reconstruction of a transcriptome from RNA-seq reads. *Nat. Biotechnol.* **33**, 290–295 (2015).
9. R Core Team. R: A Language and Environment for Statistical Computing. *R Foundation for Statistical Computing*. (2017).
10. Robinson, M. D., McCarthy, D. J. & Smyth, G. K. edgeR: a Bioconductor package for differential expression analysis of digital gene expression data. *Bioinformatics* **26**, 139–140 (2010).
11. Xu, L. *et al.* OrthoVenn2: a web server for whole-genome comparison and annotation of orthologous clusters across multiple species. *Nucleic Acids Res.* **47**, W52–W58 (2019).
12. Li, L., Stoeckert, C. J. & Roos, D. S. OrthoMCL: identification of ortholog groups for eukaryotic genomes. *Genome Res.* **13**, 2178–2189 (2003).
13. Buchfink, B., Xie, C. & Huson, D. H. Fast and sensitive protein alignment using DIAMOND. *Nat. Methods* **12**, 59–60 (2015).
14. Georgiadou, A. *et al.* Comparative transcriptomic analysis reveals translationally relevant processes in mouse models of malaria. *eLife* **11**, e70763 (2022).
15. McCune, B. & Mefford, M. J. *PC-ORD: multivariate analysis of ecological data*. (MjM Software Design, 1999).
16. McCune, B. & Mefford, M. J. PC-ORD: Multivariate Analysis of Ecological Data. (1999b).
17. Korb, J. *et al.* Comparative transcriptomic analysis of the mechanisms underpinning ageing and fecundity in social insects. *Philos. Trans. R. Soc. B Biol. Sci.* **376**, 20190728 (2021).
18. Blanco, E., González-Ramírez, M. & Di Croce, L. Productive visualization of high-throughput sequencing data using the SeqCode open portable platform. *Sci. Rep.* **11**, 19545 (2021).
19. Supek, F., Bošnjak, M., Škunca, N. & Šmuc, T. REVIGO Summarizes and Visualizes Long Lists of Gene Ontology Terms. *PLOS ONE* **6**, e21800 (2011).

Supplemental Figure 1. Inter-species ortholog comparison of the top 70 one-to-one orthologs with the greatest variance in Log fold-change expression of bacteriocytes compared to body tissues for both *M. persicae* and *A. pisum*. The z-score (within species) of Log Fold Change (bacteriocyte vs body) is presented on the figure with the color legend. Each row represents a shared one-to-one ortholog between *M. persicae* and *A. pisum*. Ortholog gene names and their corresponding GO terms for both aphid species are presented to the right of each shared one-to-one ortholog. n/a indicates no GO term was annotated for that one-to-one ortholog pair.

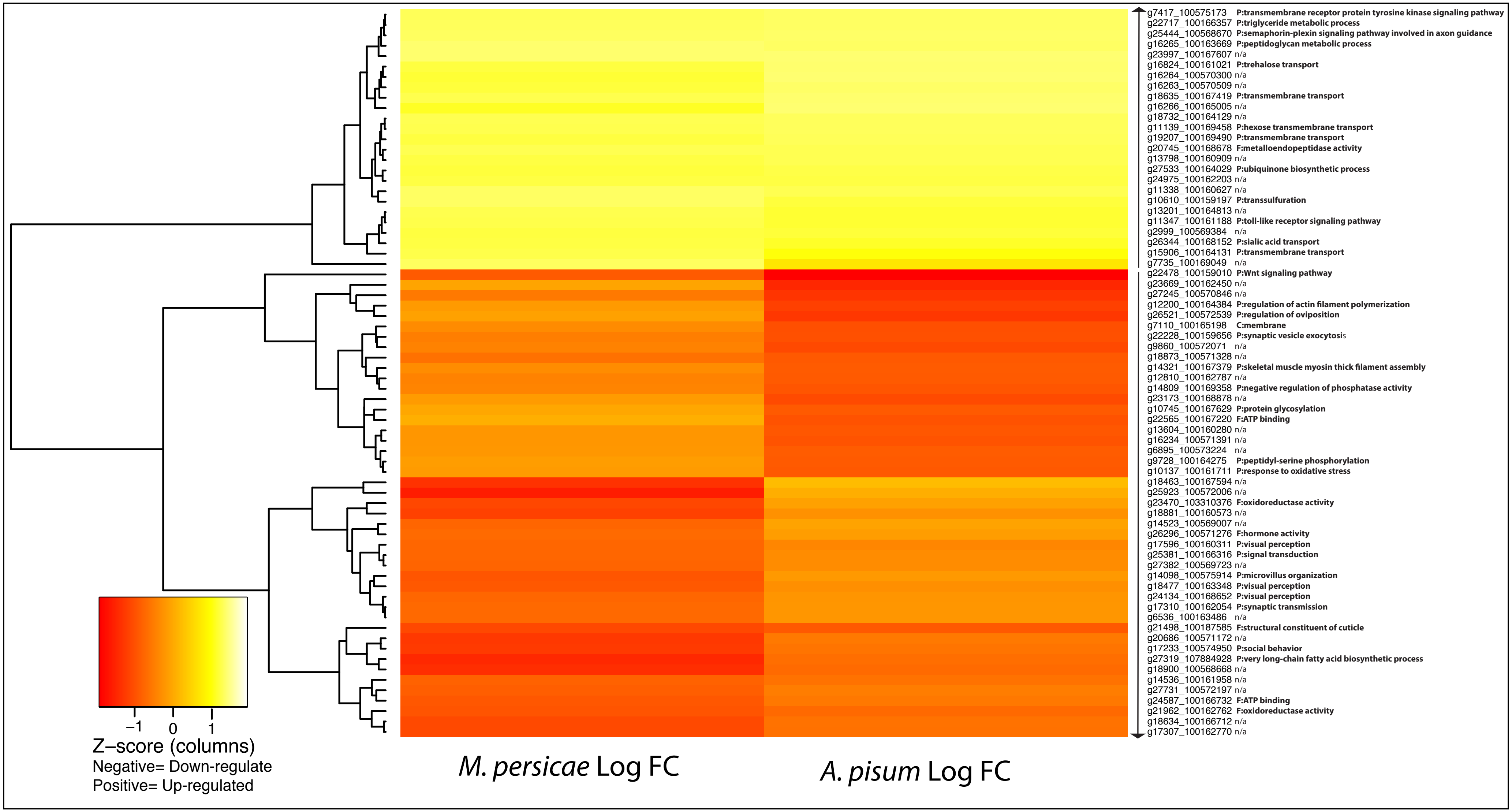

Supplemental Figure 2. Principal Component Analysis of uncorrected gene counts for both aphid species. Each point represents an independent sub-clone biological replicate (N=3; ~200 dissected and pooled aphid individuals from a species for each biological replicate/sub-clone) of either pooled bacteriocyte samples (Bac) or pooled body tissue samples without bacteriocytes (Bod). *Acyrthosiphon pisum* independent biological replicates/sub-clones are FB1, FB2, and FB3. *Myzus persicae* independent biological replicates/subclones are MP1, MP2, and MP3. The x-axis explains the most amount of variation in the data for both plots.

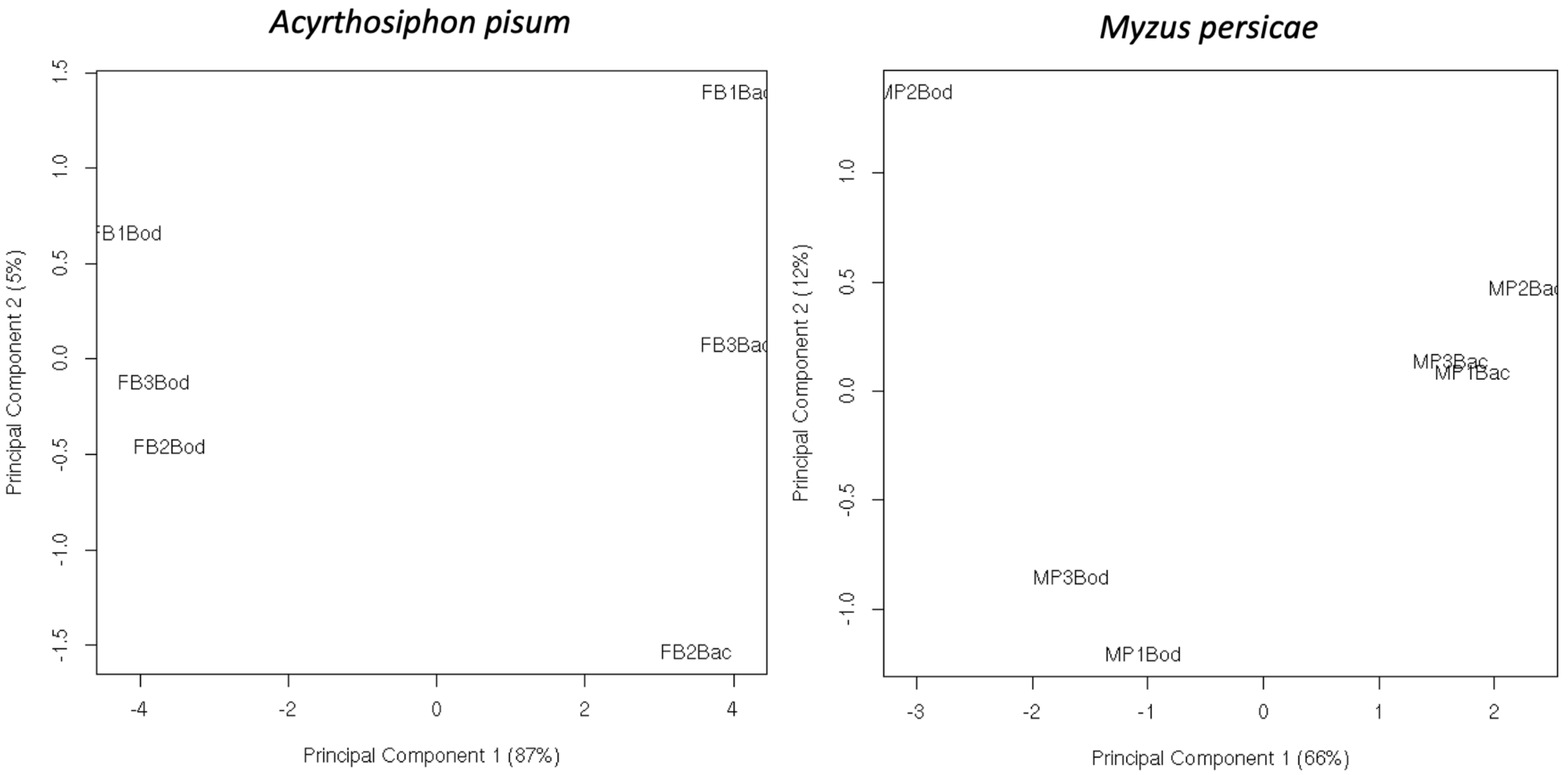

Supplement: Supplementary file 1 — Supplementary Information 1. [file 41598_2023_32291_MOESM1_ESM.pdf]
